# C‐Arm Cone Beam CT‐Guided Preoperative Microcoil Pulmonary Ground Glass Nodule Localization: Diagnostic and Surgical Advantage

**DOI:** 10.1111/1759-7714.70152

**Published:** 2025-09-03

**Authors:** Carlo Altomare, Rebecca Casati, Giuseppina Pacella, Laura Olivieri, Angelo Tirabasso, Annamaria Altomare, Luca Frasca, Filippo Longo, Pierfilippo Crucitti, Eliodoro Faiella, Bruno Beomonte Zobel, Rosario Francesco Grasso

**Affiliations:** ^1^ Unit of Diagnostic Imaging and Interventional Radiology Fondazione Policlinico Universitario Campus Bio‐Medico Rome Italy; ^2^ Unit of Diagnostic Imaging and Interventional Radiology, Department of Medicine and Surgery Università Campus Bio‐Medico di Roma Rome Italy; ^3^ Department of Occupational and Environmental Medicine, Epidemiology and Hygiene National Institute for Insurance Against Accidents at Work Rome Italy; ^4^ Unit of Gastroenterology Università Campus Bio‐Medico di Roma Rome Italy; ^5^ Unit of Thoracic Surgery Fondazione Policlinico Universitario Campus Bio‐Medico Rome Italy; ^6^ Unit of Thoracic Surgery, Department of Medicine and Surgery Università Campus Bio‐Medico di Roma Rome Italy

**Keywords:** cone beam CT, image‐guided surgery, microcoil localization, minimally invasive surgery, preoperative localization, pulmonary nodule, thoracic surgery, uniportal VATS

## Abstract

**Objective:**

This study evaluates the effectiveness and safety of C‐arm cone beam CT (CBCT)‐guided microcoil localization combined with uniportal video‐assisted thoracoscopic surgery (VATS) for the management of small, difficult‐to‐localize ground‐glass opacities (GGOs) and sub‐solid nodules in the lungs.

**Methods:**

We retrospectively analyzed data from 13 patients with single, small, peripheral, non‐subpleural GGOs or SSN. All patients underwent successful microcoil localization using CB‐CT guidance followed by uniportal VATS resection. A microcoil was positioned partly in the lung parenchyma and partly in the extra‐pleural space to assist in intraoperative localization. We evaluated the rate of correct microcoil placement and the technical success of the resection.

**Results:**

Microcoil placement was successfully performed in all patients, with an average procedure time of 28.8 ± 10.8 min. The mean nodule size was 9.9 ± 5.4 mm, and 76.9% of the nodules were classified as ground‐glass opacities. No intraparenchymal bleeding was observed, and four patients (30.8%) experienced pneumothorax, all of which were self‐limited and required no intervention or coil repositioning. The uniVATS resection success rate was 100%.

**Conclusion:**

CBCT‐guided microcoil localization, with partial placement of the coil in the extra‐pleural space, proved to be a highly effective technique for the localization and resection of small pulmonary nodules. The procedure demonstrated high accuracy, minimal complications, reduction of procedural time, and short hospital stays. Intraoperative fluoroscopy was never necessary, with a high reduction in radiation exposure for the patient and the operator. Further studies with larger populations and longer follow‐ups are needed to validate these findings.

## Graphical abstract

Detection and selection: low‐dose CT screening (within “A breath for life” program). Identification of small, peripheral, non‐subpleural GGOs or sub‐solid nodules (< 25 mm). Preoperative localization. C‐arm cone beam CT (CBCT)‐guided microcoil placement. “Push and pull” technique with distal end near nodule and proximal end in extra‐pleural space. Immediate post‐procedural CBCT confirmation. Surgical resection: same‐day uniportal VATS wedge, segmentectomy, or lobectomy: direct visual identification of coil (no intraoperative fluoroscopy required). Frozen section for diagnosis and surgical planning. Key outcomes: 100% success in localization and resection. Mean procedure time: 28.8 ± 10.8 min. Complications: 30.8% pneumothorax (all self‐limiting); no bleeding. Reduced radiation exposure for patient and operator. Mean hospital stay: 4.0 ± 1.5 days. Conclusion: CBCT‐guided microcoil localization with partial extra‐pleural placement, combined with uniportal VATS, is a safe, accurate, and efficient strategy for managing small, difficult‐to‐localize pulmonary nodules, avoiding intraoperative fluoroscopy, and minimizing complications.

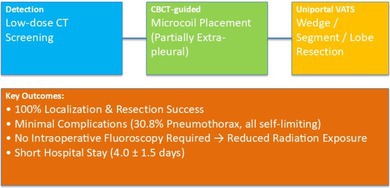

## 1 Introduction

Lung cancer is one of the most serious cancers, with the highest morbidity and mortality rates, especially among the elderly. With the significant increase in the elderly population in recent years, prevention and control of lung cancer have become increasingly crucial. Non‐small cell lung cancer (NSCLC) is the most common type of lung cancer [1].

Research has shown that the 5‐year survival rate for patients with advanced lung cancer is less than 2%; while the 5‐year survival rate for early‐stage lung cancer, detected as ground glass opacity (GGO), can be as high as 100% [2]. Therefore, early diagnosis is necessary, especially with the development of new medical technologies, such as high‐resolution CT scans, which have become the primary imaging method [3]. With these advances in diagnostics, GGO or sub‐solid nodules are often extremely small and represent a significant challenge for lung biopsies, which can even result in false negatives. Indeed, in these cases, if malignancy is strongly suspected, surgical resection is recommended to obtain a rapid diagnosis and early treatment.

Unfortunately, traditional thoracotomy is a highly invasive and traumatic surgical procedure with a high rate of postoperative complications [4]. Video‐assisted thoracoscopic surgery (VATS) is often used for excisional biopsy to minimize costs, postoperative complications, and the amount of lung tissue removed. However, this less invasive surgical procedure is frequently converted to a thoracotomy due to the inability to localize the lung nodule. The most common risk factors for this conversion are the distance from the pleural surface and the size of the nodule [5]. The key to addressing many of these issues is to localize the neoplasm before performing VATS. Techniques such as CT‐guided placement of microcoils, markers, or dyes can be employed to mark the precise location of the nodules preoperatively. These markers guide the surgeon during VATS, ensuring accurate and efficient resection of the target tissue. Additionally, intraoperative imaging techniques, such as fluoroscopy or ultrasound, can assist in real‐time localization and verification of the nodules [6].

By integrating these advanced localization techniques with VATS, the accuracy of nodule resection improves significantly, enhancing the overall efficacy of the procedure. This approach not only facilitates early and precise diagnosis but also optimizes therapeutic outcomes, contributing to better patient prognosis and quality of life [7]. The continuous evolution of imaging and surgical technologies promises further improvements in the management of lung nodules, paving the way for even less invasive and more effective interventions in the future.

The objective of this study is to evaluate the effectiveness and safety of C‐arm cone beam CT (CBCT)‐guided microcoil localization, followed by uniportal VATS resection for the management of small, difficult‐to‐localize pulmonary nodules.

## 2 Materials and Methods

We retrospectively evaluated data from 13 patients (9 females and 4 males) affected by single, small, peripheral, non‐subpleural pulmonary GGOs or sub‐solid nodules, who underwent successful uniportal wedge resection after CT‐guided microcoil localization at our institution from April 2022 to April 2024.

All nodules were diagnosed through the patients' participation in the “a breath for life” screening program.

“A breath for life” is a program that offers to all smokers between the ages of 50 and 75 or those who have stopped smoking less than 10 years ago, the opportunity to check the health of their lungs free of charge using a low‐dose CT scan.

The mean patient age was 64.2 ± 8.9 years (age range, 52–78 years).

The average body mass index (BMI) of the patients was 25.6 ± 5.5 kg/m^2^ (range, 17.5–34.4 kg/m^2^).

All patients had peripheral lesions characterized by dimensional growth or suspicious morphology. The inclusion criteria were: lesion unfit for CT‐guided FNCB for small dimensions; and lesion with a previous non‐diagnostic biopsy.

The only important exclusion criterion was a lesion depth > 5 cm; which would result in significantly greater lung parenchyma removal.

Pre‐operative imaging included a combination of high‐resolution CT and PET‐CT, with 38.5% of the cases localized in the right upper lobe and 46.1% in the left lung.

### 2.1 Microcoil Placement

All procedures were performed under sterile conditions and local anesthesia, using CBCT‐guidance and the IMACTIS electromagnetic navigation system. This system, by visualizing the needle path, allowed for improved accuracy in the lung marker placement. A microcoil, typically Penumbra 5 mm × 12 cm, was positioned at the level of the pulmonary nodule using an ECOJET 150 needle.

The coil is positioned with a “push and pull” technique to release the distal end near the pulmonary nodule and the proximal end into the extra‐pleural space.

**FIGURE 1:**
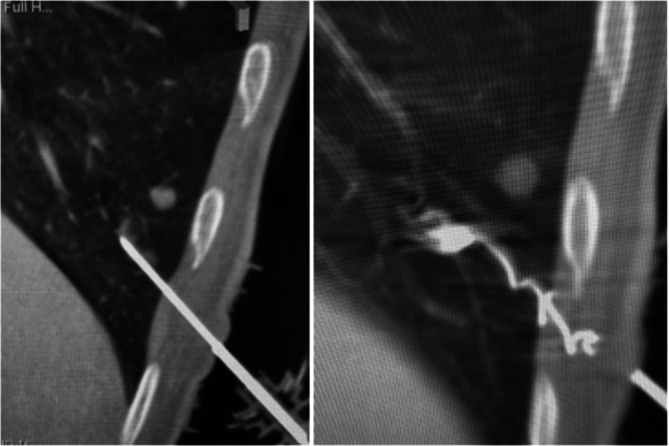
Final CT scan image showing the proximal extremity microcoil accurately positioned within the lung parenchyma at the level of the suspicious nodule and the distal extremity in the extra‐pleural space. The coil's placement is confirmed post‐intervention, with 31.0 mm from the pleura, ensuring precise localization for subsequent surgical resection.

A postprocedural CB‐CT scan is obtained to assess the final position of the microcoil to the nodule (Figure 1). Upon completion, the patient was transferred to the operating room for surgical intervention.

### 2.2 Surgical Approach

**FIGURE 2:**
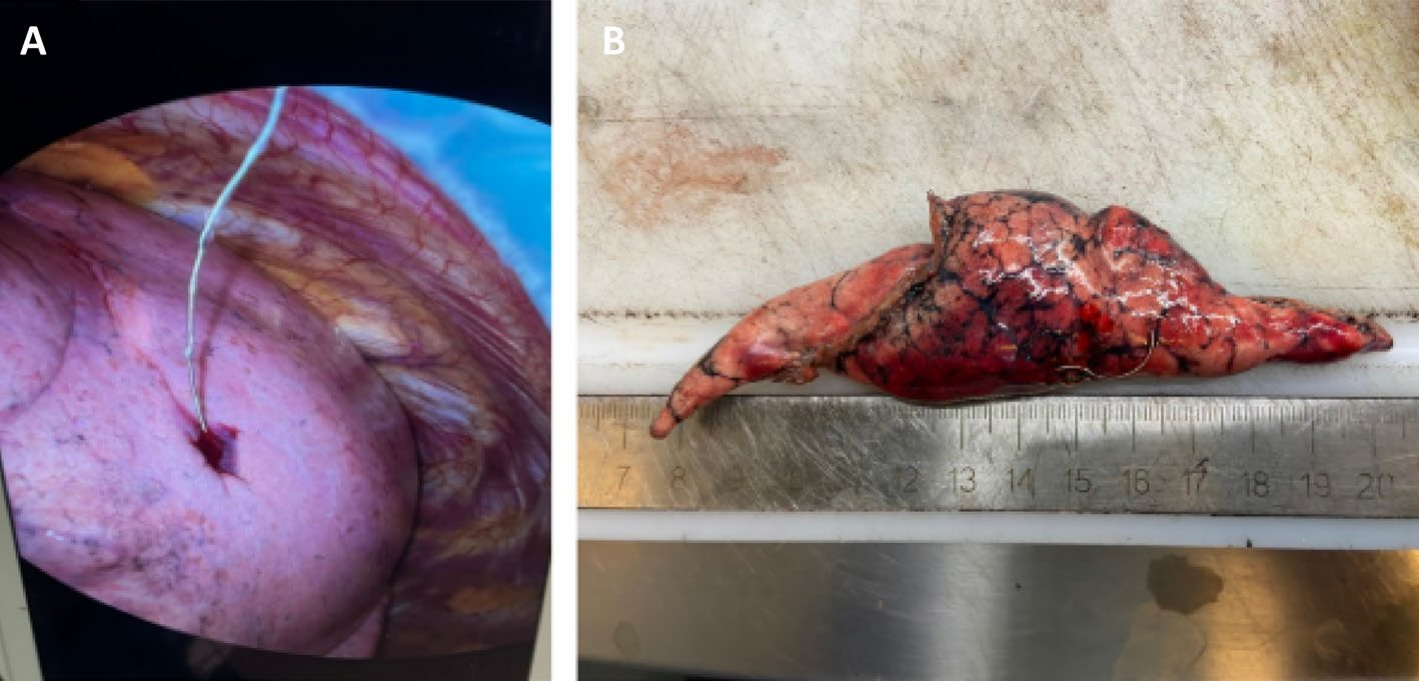
(A) The microcoil is visualized during the uniVATS, positioned to guide the resection of the pulmonary nodule. (B) Microcoil after resection into the lung specimen.

The surgical procedure is performed on the same day the microcoil is placed radiologically. After inducing lung collapse, the visceral pleura was carefully inspected until direct visualization of the coil was confirmed (Figure 2) making the use of intraoperative fluoroscopy unnecessary.

Once the marked lesion was identified, a large‐ring forceps was used to excise the lesion along with 2 cm of surrounding lung parenchyma, performing a wedge resection. All lung specimens underwent frozen section examination to evaluate both the excision and the nature of the lesions (benign vs. malignant). If malignancy was confirmed, we proceeded with a major lung resection, choosing between segmentectomy and lobectomy based on the lesion's location and the percentage of the solid component (segmentectomy if the solid component was less than 50% of the ground‐glass opacity). The primary endpoints of the study were the rate of intraoperative localization of marked lesions and the success rate of uniVATS resections.

## 3 Results

The pulmonary nodules had an average size of 9.9 ± 5.4 mm (range, 6–25 mm). Most of the nodules were of the “ground glass” type (10; 76.9%), and three were mixed type (23%). The mean distance from the visceral pleura was 10.2 ± 7.2 mm (range, 1–21 mm).

The microcoil placement was successfully performed in all patients. The time required for the CT‐guided microcoil positioning averaged 28.8 ± 10.8 min (range, 17–58 min). In the two longest procedures we recorded, one finding was a high BMI > 30. There are no further objective data responsible for the wide range of the average coil placement time. A relatively longer time margin could be related to patient compliance and the experience of the operator, but this data are not quantifiable.

No intraparenchymal bleeding was registered. There were four cases of pneumothorax following microcoil placement, none of which required aspiration or chest tube drainage.

Subsequently, a uniportal VATS was performed for each patient to locate the pulmonary nodule. In 10 cases (76.9%), the microcoil remained in place, while in 3 cases (23%, cases 2, 9 and 10 of Table 1) the microcoil was displaced completely into the extra‐pleural space. In these cases, visualization of the parietal/visceral pleural puncture site allowed us to localize the target lesion; specifically, the puncture site was visible thanks to the presence of a small adjacent parenchymal hematoma. No palpation was required; neither instrument guidance nor any other assistance.

**TABLE 1:** Data regarding nodule localization and the distance between nodule‐pleura and skin‐pleura of all the patients. Cases were the microcoil has been dispaced hare highlighted in grey. Abbreviations: Right Upper Lobe (RUL); Left Upper Lobe (LUL), Right Lower Lobe (RLL), Left Lower Lobe (LLL).

Case	BMI	Nodule localization	Distance nodule‐pleura	Distance skin‐pleura
1	26.42	RUL	9	9
2	32.81	RUL	12	40
3	21.60	LUL	6	6
4	30.09	LUL	19	19
5	23.67	RLL	4	4
6	23.87	RUL	12	20
7	21.53	RLL	4	15
8	28.90	LUL	4	4
9	26.58	RUL	5	10
10	21.90	LUL	10	12
11	28.98	LLL	27	27
12	22.64	LUL	7	12
13	23.64	RUL	8	15

One of the three patients with microcoil displacement had an elevated BMI > 30 kg/m^2^ and, consequently, the distance between the pleura and the skin was more than 4 cm. In two patients, the lesion was in the upper right and left lobes, and in the third patient in the right lower lobe. Two of the three nodules were located at a depth of more than 10 mm from the pleura; only one of these nodules was located at less than 5 mm from the pleura. None of these patients had any pulmonary disease or other significant risk factors. All the data regarding nodule localization and the distance between nodule‐pleura and skin‐pleura are reported in Table 1.

Histopathological examination of the resected specimens revealed adenocarcinoma in the majority of patients (9; 69.2%), exhibiting various growth patterns, including lepidic and papillary subtypes. Two cases were diagnosed as sarcomas (15.4%), one as an inflammatory granuloma (7.7%), and one as a localization of Hodgkin's lymphoma (7.7%). The nodules that were found to be sarcomas were both new and therefore suspicious. Furthermore, one of the two patients had a history of monophasic synovial sarcoma in the left plantar region, treated with neoadjuvant radiotherapy and operated on a year earlier. On CT, both nodules appeared small, solid, and with regular margins. In the case of lymphoma, the patient was already known to have this disease; the nodule appeared subsolid with irregular margins on PET‐CT images.

Regarding the surgical procedure, eight patients underwent wedge resection of the lung, three underwent segmentectomy, and two required lobectomy. The mean hospital stay was 4.0 ± 1.5 days (range, 3–8 days). The successful uniVATS resection rate was 100%.

## 4 Discussion

The management of small pulmonary nodules, particularly ground‐glass opacities (GGOs) and sub‐solid nodules, poses significant challenges due to their subtle nature and often undetectable consistency. With the increasing implementation of lung cancer screening programs, the detection of these nodules has become more frequent, especially in asymptomatic patients [8]. While their early detection is critical, the limitations of traditional biopsy techniques, particularly for GGOs, require alternative approaches for accurate diagnosis and treatment. Various techniques have been explored to improve the accuracy of nodule identification, each offering its advantages and disadvantages.

Our results demonstrated a 100% success rate in the localization and resection of the nodule using this modified approach.

Only three patients showed a microcoil displacement following the pneumothorax induction during the VATS; in this study group, none of these conditions—lobe location, BMI, lesion depth, or any shared risk factors—seem to be involved in this surgical complication.

Despite this, it is possible that the coil dislocation was due to the reduced intraparenchymal portion of the coil compared to that left in the extra‐pleural space. Further studies are needed to confirm this hypothesis. Moreover, the three coils were released during the first procedures performed, likely due to the operator's lack of experience.

Therefore, it was interesting to observe that the coil dislocation didn't impact any delay or increased difficulty during surgery, particularly regarding operative time or localization accuracy.

Despite 30.8% pneumothorax following microcoil placement, none of these cases required chest drainage and were self‐limited without needing an intervention. No patient experienced intraparenchymal bleeding.

Unlike most studies in the literature, which use conventional computed tomography (CT) guidance for microcoil placement, in our study we used CB‐CT guidance. CB‐CT provides high‐resolution, three‐dimensional images similar to CT images but also allows real‐time visualization of the microcoil using fluoroscopy, improving the accuracy of coil placement [9, 10]. This imaging modality allows for immediate assessment and correction during the procedure, potentially offering advantages in reducing overall procedure time and radiation exposure compared to conventional CT scans [9, 10], even if, in this study, comparative data are not present. The localization success rate of our procedures was 100%, comparable to the results obtained from previous studies [9, 10].

Furthermore, unlike classic approaches where the microcoil is placed entirely within the lung parenchyma, we left the microcoil partly within the lung and partly extended into the EXTRA‐pleural space [11]. This technique offers a significant intraoperative advantage; the portion of the microcoil protruding into the pleural space serves as a direct visual marker during uniportal VATS, facilitating rapid and accurate localization of the target nodule without the need for intraoperative fluoroscopy or additional techniques, reducing exposure to ionizing radiation in the operating room for both the patient and the operators compared to other studies [9, 10].

These results support the safety profile of this technique, with lower complication rates compared to other marking devices such as hook wire and lipiodol [12]. Another technique involves the use of hydrogel plugs. While these devices have shown promising results with a low complication rate, they have been associated with a higher incidence of marker dislocation compared to microcoils [13, 14].

The mean time for coil placement (28.8 ± 10.8 min) was reasonable and the hospital stay was 4.0 ± 1.5 days, comparable to standard results for minimally invasive thoracic surgery.

However, as our study did not include a control group or a direct comparison with CT fluoroscopic microcoil placement, no definitive conclusion can be drawn from our results regarding superiority between the two techniques.

A limitation of this technique is the high cost of the coil; however, this expense has been shown to be offset by savings in operative time, disposable costs, and the avoidance of additional biopsy procedures needed to establish the pathological diagnosis of the nodule [7]. Performing both microcoil placement and surgical resection during the same hospitalization is useful to prevent coil dislodgement, reduce healthcare costs, and minimize patient stress.

## 5 Conclusion

In conclusion, we believe that combining CB CT‐guided microcoil localization—with the microcoil placed within the parenchymal lung and partly in the extra‐pleural space—along with uniportal VATS, represents a highly effective strategy for managing small and difficult‐to‐localize pulmonary nodules. This approach not only improves nodule resection accuracy but also reduces exposure to ionizing radiation in the operating room for both the patient and the operators, enhancing safety. Additionally, it is an intuitive method that accelerates the surgical procedure, reducing intraoperative procedural time while maintaining a low complication rate, making it a valuable tool in modern thoracic surgery. A limitation of our study is the relatively small sample size, which may affect the generalizability of our results, as well as the short follow‐up period, which limits our ability to assess long‐term outcomes such as recurrence rates and overall survival. Future studies are necessary to validate these findings and determine whether these results can be replicated in a larger population.

## Author Contributions

Conceptualization, C.A., G.P., F.L., R.F.G.; methodology, C.A., R.C., L.O., L.F., P.C.; formal analysis, C.A., L.O., A.T., G.P., F.L.; investigation, C.A., R.C., G.P., L.F., F.L.; data curation, C.A., L.O., R.C., A.T., L.F., F.L.; writing – original draft preparation, C.A., A.A., L.F., F.L., P.C., E.F.; writing – review and editing, C.A., A.A., P.C., E.F., B.B.Z., R.F.G.; supervision, P.C., E.F., B.B.Z., R.F.G. All authors have read and agreed to the published version of the manuscript.

## Conflicts of Interest

The authors declare no conflicts of interest.

## Data Availability

Data are available on request to the corresponding author.
